# Bromination and Diazo-Coupling of Pyridinethiones; Microwave Assisted Synthesis of Isothiazolopyridine, Pyridothiazine and Pyridothiazepines

**DOI:** 10.3390/molecules17066930

**Published:** 2012-06-06

**Authors:** Ayman M. S. Youssef, Mohamed E. Azab, Mohamed M. Youssef

**Affiliations:** 1 Department of Chemistry, Faculty of Science, Fayoum University, Fayoum 63514, Egypt; 2 Department of Chemistry, Faculty of Science, El-Jabal El-Gharby University, Mezda, Gheryan 65096, Libya; 3 Department of Chemistry, Faculty of Science, Taif University, Taif 21974, Saudi Arabia

**Keywords:** microwave, bromination, diazo-coupling, pyridinecarboxamide, isothiazolopyridine, pyridothiazine, pyridothiazepine

## Abstract

Isothiazolopyridines, pyridothiazines and pyridothiazepines are important compounds that possess valuable biological activities. This paper reports on the synthesis of these compounds using both conventional chemical methods and modern microwave techniques. 3-Bromo-6-hydroxy-4-methyl-2-thioxo-2,3-dihydropyridine-3-carboxamide, 5-arylazo-6-hydroxy-4-methyl-2-thioxo-1,2-dihydropyridine-3-carboxamides, 3,5-bis-arylazo-6-hydroxy-4-methyl-2-thioxo-2,3-dihydropyridine-3-caboxamide, 4-methyl-2,3,6,7-tetra-hydroisothiazolo[5,4-b]-pyridine-3,6-dione, 2,2'-(methylene-bis-(sulfanediyl))bis(4-methyl-6-oxo-1,6-dihydropyridine-3-carboxamide), 2-hydroxy-5-methyl-4*H*-pyrido[3,2-e][1,3]-thiazine-4,7(8*H*)-dione and 2-arylmethylene-8-hydroxy-6-methyl-2,3,4,5-tetrahydropyrido-[3,2-f][1,4]thiazepine-3,5-diones have been prepared from 6-hydroxy-4-methyl-2-thioxo-2,3-dihydropyridine-3-carboxamide. Some of these compounds were prepared using microwave-assisted reaction conditions, that provided higher yields in shorter times than the conventional methods.

## 1. Introduction

In continuation of our research groups’ work in the field of synthetic organic chemistry [1,2,3,4,5]; we would like to report here on the annulation of isothiazole, 1,3-thiazine and 1,4-thiazepine ring systems to pyridine. Promising biological activities are reported in the literature concerning the targeted ring systems: Isothiazolo[5,4-b]pyridines could be used as analgesics [6,7] and as CNS and antimicrobial agents [8], pyrido[3,2-e][1,3]thiazines could be used as CNS and antioxidant agents [9] and as analgesic agents [10] and pyrido[3,2-f]-[1,4]-thiazepines were used as calcium antagonists in both cardiac and smooth muscles [11] and as channel blockers for treatment of cardiovascular diseases [12]. Microwave-assisted technique offer several advantages over conventional methods of synthesis. Reduced reaction times [13,14,15], less effects on the environment and better reaction yields are some of the common advantages of using microwave irradiation. In the present research project, we used both the microwave technique as well as conventional methods to prepare the targeted compounds with expected biological activity.

## 2. Results and Discussion

**Scheme 1:**
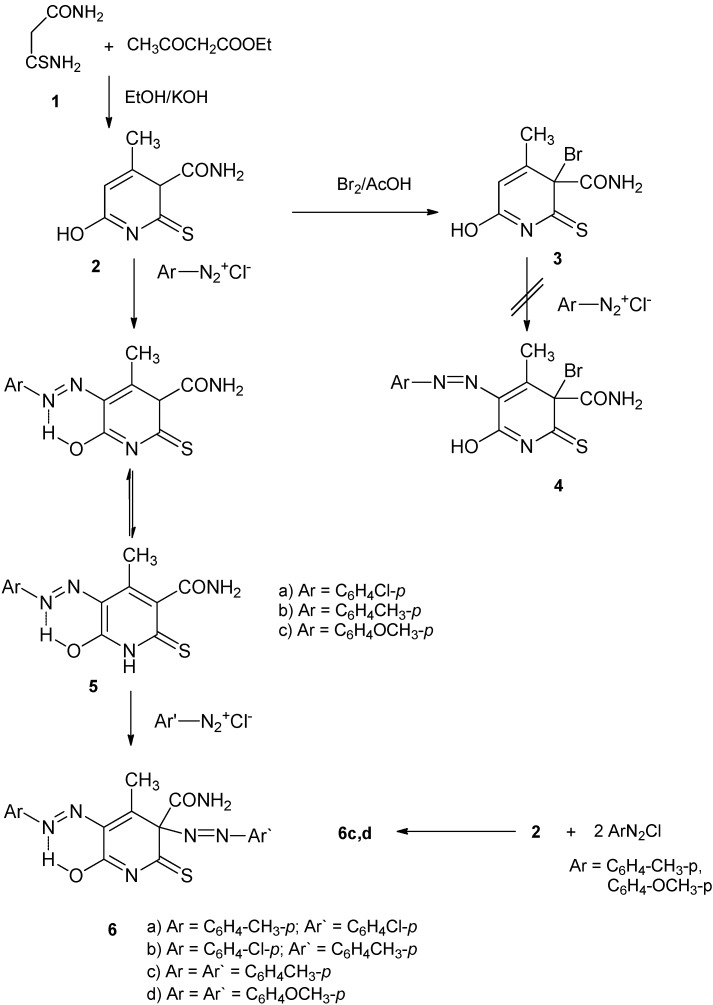
Bromination and diazo coupling of the pyridinethione derivative **2**.

The starting compound 6-hydroxy-4-methyl-2-thioxo-2,3-dihydropyridine-3-carboxamide (**2**) was prepared from 3-amino-3-thioxopropanamide [monothiomalonamide, (**1**)] [16] and ethyl acetoacetate according to the literature procedure [17] (Scheme 1). Bromination of **2** with bromine in acetic acid yielded 3-bromo-6-hydroxy-4-methyl-2-thioxo-2,3-dihydropyridine-3-carboxamide (**3**).

Our preference for structure **3** for the reaction product was based on the comparison of both the ^1^H-NMR and ^13^C-NMR spectra (DMSO-d_6_) of **2** and **3**. In the ^1^H-NMR spectrum of **2**, there are two signals at δ 5.24 and δ 6.10 ppm corresponding to the pyridine-H3 and pyridine-H5, respectively. The ^1^H-NMR spectrum of the reaction product revealed a signal at δ 6.12 ppm corresponding to the pyridine-H5. The ^13^C-NMR spectrum of **2** showed two signals at δ 71.5 and δ 112.1 ppm corresponding to C3 and C5, respectively. The reaction product showed two signals at δ 91.3 and 111.8 ppm corresponding to the same carbon atoms. The downfield shift of C3 indicates that bromination took place on this carbon atom, favouring structure **3** for the reaction product. The reaction product assigned structure **3** was most probably due to an initial protonation on the amide carbonyl that leads to higher activity of position 3 in compound **2**, and so the formation of the kinetically controlled product **3** is favoured. 

Attempts to couple compound **3** with benzenediazonium chloride to produce **4** have all failed, most probably due to lack of aromaticity in compound **3**. In contrast to the reaction of bromine with compound **2**, the latter compound coupled with some arenediazonium salts at position 5 to give the monoarylazo derivatives. Thus, treatment of **2** with equimolar amounts of some arenediazonium salts yielded 5-arylazo-6-hydroxy-4-methyl-2-thioxo-1,2-dihydropyridine-3-carboxamides **5a–c** (Scheme 1).

Structure **5** for the reaction product was based on a comparison between the ^1^H-NMR spectra of **2** and **5a** that revealed the disappearance of the signals at δ 5.24 & 6.10 ppm in **5a**. The disappearance of the second one indicates that substitution takes place at position 5, while disappearance of the first one is due to tautomerization and appearance of a D_2_O exchangeable signal at δ 12.86 ppm. Also, the ^13^C-NMR spectrum of compound **2** showed signals at 71.5 and 112.1, corresponding to C-3 and C-5, respectively. The same two carbons in **5a** showed signals at 72.3 and 123.8, respectively. The downfield shift of C-5 signal indicates that substitution took place at this carbon atom. As further evidence, the monoarylazo derivatives **5a,b** could be coupled with some arenediazonium salts to give bisarylazo derivatives (see below). An alternative *ipso* attack structure would have no aromaticity and would not undergo further coupling.

The monoarylazo derivatives **5a,b** coupled with some arenediazonium salts to give 3,5-bis-arylazo-6-hydroxy-4-methyl-2-thioxo-2,3-dihydropyridine-3-caboxamides **6a,b** (Scheme 1). Bisarylazo derivatives **6c,d** could be synthesized directly from **2** by treatment with double amount of the diazonium salt (Scheme 1).

**Scheme 2:**
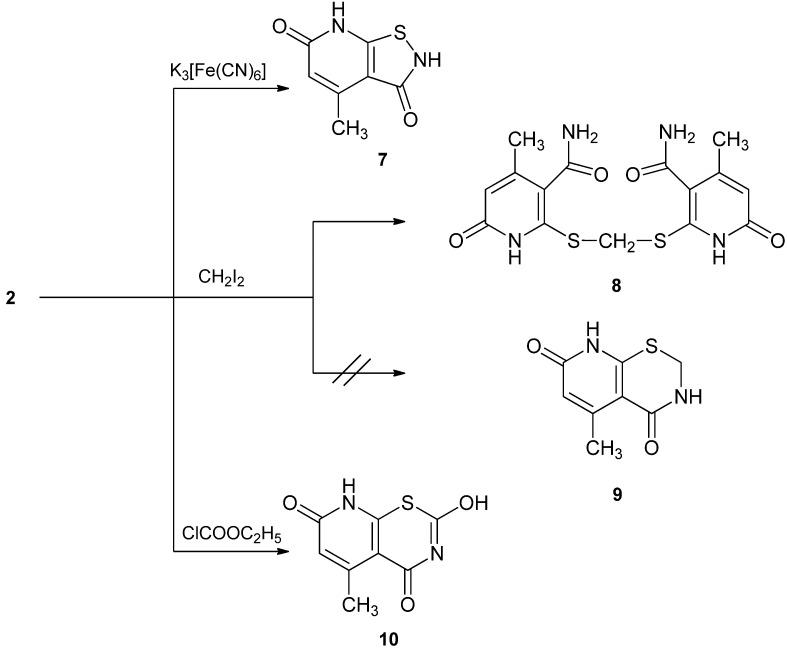
Formation of isothiazolopyridine, methylene-bis-sulfanediyl-bis-pyridinone and pyridothiazine.

Next, we aimed to annulate five, six and seven-membered rings to the starting pyridine. A five-membered ring is annulated by oxidation of **2** with potassium ferricyanide in ethanolic potassium hydroxide solution, which led to the formation of 4-methyl-2,3,6,7-tetrahydroisothiazolo[5,4-b]-pyridine-3,6-dione (**7**, Scheme 2).

To annulate a six-membered ring to the starting pyridinethione derivative, compound **2** was allowed to react with diiodomethane in ethanolic potassium hydroxide solution. The reaction product was proved to be 2,2'-(methylene-bis(sulfanediyl))bis(4-methyl-6-oxo-1,6-dihydropyridine-3-carboxamide) (*i.e*., **8** instead of the expected pyrido[3,2-e][1,3]thiazine **9** (Scheme 2).

The IR spectrum of **8** displayed absorption bands at 3358 and 3171 cm^−1^ (NH) and 1681 and 1635 cm^−1^ (CO). Its ^1^H-NMR spectrum (DMSO-d_6_) showed signals at δ 2.22 ppm (s, 6H, 2CH_3_), 4.90 (s, 2H, CH_2_), 6.27 (s, 2H, pyridine-H-5), 7.52–7.77 (brs, 4H, 2 NH_2_, D_2_O exchangeable) and 11.00 (s, 2H, 2 × NH, D_2_O exchangeable). The mass spectrum of **8** showed the molecular ion peak at *m/z* 380 (1.5%), corresponding to C_15_H_16_N_4_O_4_S_2_. The base peak appeared at *m/z* 53. 

Treatment of **2** with ethyl chloroformate, in ethanol in the presence of sodium ethoxide, led directly to the formation of 2-hydroxy-5-methyl-4*H*-pyrido[3,2-e][1,3]thiazine-4,7(8*H*)-dione (**10**, Scheme 2).

**Scheme 3:**
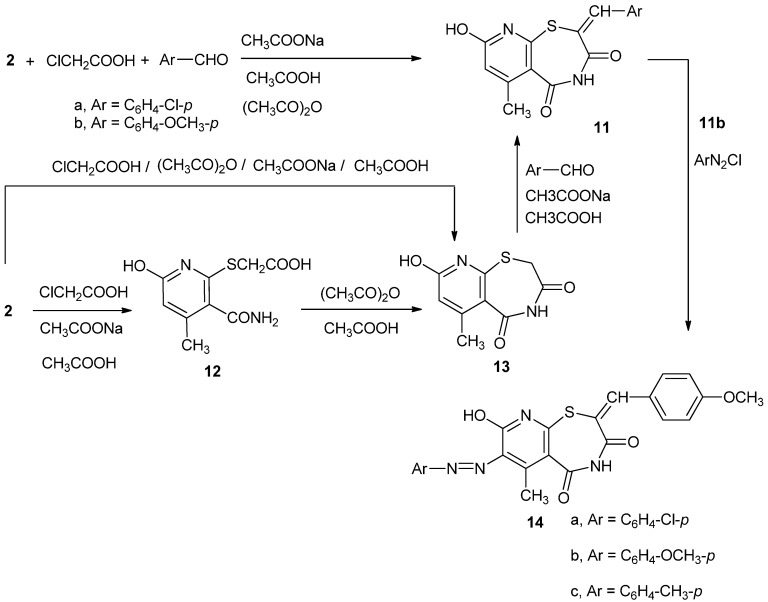
Formation of pyridothiazepine derivatives.

Heating under reflux compound **2** with chloroacetic acid and an appropriate aromatic aldehyde, in acetic acid/acetic anhydride mixture in the presence of anhydrous sodium acetate, gave directly 2-arylmethylene-8-hydroxy-6-methyl-2,3,4,5-tetrahydropyrido[3,2-f][1,4]thiazepine-3,5-diones **11a,b**, as shown in Scheme 3.

The selection of structure **11** was based firstly on the ^1^H-NMR spectrum of the reaction product that showed two types of exchangeable protons at δ 7.92 and 11.10 ppm. Secondly, structure **11** behaves like a typical 2-hydroxypyridine and couples with arenediazonium salts to give the 7-arylazoderivatives **14**, (see below). Moreover, compounds **11a,b** could also be synthesized in a stepwise fashion. Thus, heating under reflux compound **2** with chloroacetic acid in acetic acid solution in the presence of sodium acetate, yielded 2-[(3-carbamoyl-6-hydroxy-4-methylpyridin-2-yl)thio]acetic acid (**12**, Scheme 3).

Heating compound **12** in acetic acid/acetic anhydride solution, gave 8-hydroxy-6-methyl-2,3,4,5-tetrahydropyrido[3,2-f][1,4]thiazepine-3,5-dione (**13**, Scheme 3). Compound **13** could also be directly obtained from compound **2** by heating with chloroacetic acid in a mixture of acetic acid and acetic anhydride at 100 °C in the presence of anhydrous sodium acetate. Compound **13** condensed with *p*-chlorobenzaldehyde by heating under reflux in acetic acid in the presence of anhydrous sodium acetate to give **11a**, with identical m.p., m.m.p., and IR data (Scheme 3).

Compound **11b**, as a typical 2-hydroxpyridine, coupled with arenediazonium salts to give 7-arylazo-2-(p-methoxyphenylmethylene)-8-hydroxy-6-methyl-2,3,4,5-tetrahydropyrido[3,2-f][1,4]-thia-zepine-3,5-diones **14a–c**, (Scheme 3).

Our research group has recently [17,18,19] been interested in performing synthesis of some heterocyclic compounds under environmentally friendly, time saving microwave-assisted conditions. Accordingly, we re-synthesized the previously described compounds **8**, **10**, **11a**,**b**, **12** and **13** under microwave conditions, aiming to increase reaction yields and reduce the reaction times. The results of these preparations indicated that reaction yields were increased by 17–23% compared to the conventional conditions. Reaction times were also significantly reduced. Table 1 summarizes the benefits of using microwave conditions for the synthesis of the above-mentioned compounds.

**Table 1:** Comparison between traditional methods and microwave assisted methods of synthesis of compounds **10**, **12**, **13a**, **13b**, **15** and **16**.

Compound no.	Reaction Yield %		Reaction Time	
	Microwave	Conventional Method	Microwave	Conventional Method
**10**	90	68	5 min	2 h
**12**	92	75	30 min	24 h
**13a**	93	73	10 min	5 h
**13b**	88	71	10 min	5 h
**15**	92	74	5 min	3 h
**16**	66	43	5 min	3 h

## 3. Experimental

### 3.1. General

Melting points were determined in open glass capillaries on a Gallenkamp melting point apparatus and are uncorrected. IR spectra (KBr discs) were recorded on a Shimadzu FTIR-8201PC spectrophotometer. ^1^H-NMR and ^13^C-NMR spectra were recorded on a Varian Mercury 300 MHz and Varian Gemini 200 MHz spectrometers using TMS as an internal standard and DMSO-d_6_ as solvent. Chemical shifts were expressed as δ (ppm) units. Mass spectra were recorded at 70 eV on a Shimadzu GCMS-QP1000EX using an inlet type injector. All reactions were followed by TLC (silica gel, aluminum sheets 60 F254, Merck). The Microanalytical Center of Cairo University performed the microanalyses. Microwave reactions were performed with a Millstone Organic Synthesis Unit (MicroSYNTH with touch control terminal) with a continuous focused microwave power delivery system in a pressure glass vessel (10 mL) sealed with a septum under magnetic stirring. The temperature of the reaction mixture was monitored using a calibrated infrared temperature control under the reaction vessel, and control of the pressure was performed with a pressure sensor connected to the septum of the vessel. 3-Amino-3-thioxopropanamide [monothiomalonamide (**1**)] and 6-hydroxy-4-methyl-2-thioxo-2,3-dihydropyridine-3-carboxamide (**2**) were prepared according to literature procedures [16,17]. Reported ^1^H-NMR and ^13^C-NMR of **2** [14] are given for comparison: ^1^H-NMR: 2.30 (s, 3H, CH_3_), 3.85 (br s, 1H, OH, D_2_O exchangeable), 5.24 (s, 1H, H-3), 6.10 (s, 1H, H-5), 7.40 (br s, 2H, NH_2_, D_2_O exchangeable); ^13^C-NMR: 20.3 (CH_3_), 71.5 (C-3), 112.1 (C-5), 137.3, 162.3 (sp2 C), 173.1 (CO), 188.0 (CS).

### 3.2. 3-Bromo-6-hydroxy-4-methyl-2-thioxo-1,2,3,6-tetrahydropyridine-3-carboxamide *(**3**)*

To a solution of **2** (2.84 g, 0.01 mole) in glacial acetic acid (50 mL), bromine (0.5 mL, 0.01 mole) was added dropwise with stirring at room temperature in sunlight. The mixture was then stirred at water bath for two hours and then diluted with cold water (30 mL). The resultant crude product thus precipitated was collected by filtration, washed with water, dried and crystallized from dilute dimethylformamide to afford **3** in 73% yield as pale brown crystals; m.p. 310 °C; IR ν: 3100–2250 cm^−1^ (OH, NH, hydrogen bond), and 1689 cm^−1^ (CO); ^1^H-NMR: 2.42 (s, 3H, CH_3_), 6.12 (s, 1H, pyridine-H), 11.91 (broad s, 3H, NH_2_, OH, D_2_O exchangeable); ^13^C-NMR: 13.5 (CH_3_), 91.3 (C-3), 111.8 (C-5), 131.5 (C-4), 158.9 (C-6), 171.1 (CO), 182.3 (CS); Mass spectrum *m/z*: 264 (49%), 262 (48.5%), 182 (100.0%), 154 (20.8%) and 99 (19%); Anal. Calcd for C_7_H_7_BrN_2_O_2_S: C, 31.95%; H, 2.68%; Br, 30.37%; N, 10.65%; S, 12.19%. Found: C, 32.20%; H, 2.50%; Br, 30.70%; N, 10.40%; S, 12.40%. 

### 3.3. 5-Arylazo-6-hydroxy-44-methyl-2-thioxo-1,2-dihydropyridine-3-carboxamides ***5a–c***

General procedure: To a cold solution of **2** (1.84 g, 0.01 mole) in pyridine (50 mL), containing 0.3 g potassium hydroxide, the arenediazonium chloride (0.01 mole) [prepared by adding concentrated hydrochloric acid (3 mL) to aromatic amine (0.01 mol) at 0 °C and treating the resulting hydrochloride with a cold solution of sodium nitrite (0.69 g, 0.01 mol) in water (5 mL)] was added dropwise with stirring at 0 °C. The coupling mixture was stirred at room temperature for two hours and then diluted with water (30 mL). The resultant crude product thus precipitated was collected by filtration, washed with water, dried and crystallized from the proper solvent.

*5-(p-Chlorophenylazo)-6-hydroxy-4-methyl-2-thioxo-1,2-dihydropyridine-3-carboxamide* (**5a**). Crystallized from dioxane in 93% yield; m.p. 270 °C; IR ν: 3580–2122 (NH, OH) and 1651 (CO). ^1^H-NMR: 2.22 (s, 3H, CH_3_), 7.48–7.68 (m, 6H, 4 aromatic protons and NH_2_, D_2_O exchangeable), 12.86 (s, 1H, NH, D_2_O exchangeable), 13.87 (s, 1H, hydrogen bonded OH, D_2_O exchangeable); ^13^C-NMR: 13.4 (CH_3_), 72.3 (C-3), 123.8 (C-5), 129.8, 132.2, 134.5, 137.3, 146.0, 160.4 (Ar-H + pyridine C-4, C-6), 172.7 (CO), 185.3 (CS); Mass spectrum *m/z*: 324 (15.1%), 322 (44.9%), 287 (24.5%), 276 (6.2%), 198 (3.8%), 111 (80%) and 60 (100.0%); Anal. Calcd for C_13_H_11_ClN_4_O_2_S: C, 48.37%; H, 3.44%; Cl, 10.98%; N, 17.36%; S, 9.93%. Found: C, 48.60%; H, 3.50%; Cl, 11.20%; N, 17.40%; S, 10.20%.

*6-Hydroxy-4-methyl-2-thioxo-5-(p-tolyldiazenyl)-1,2-dihydropyridine-3-carboxamide* (**5b**). Crystallized from dimethylformamide in 87% yield; m.p. 308 °C; IR ν: 3433–2160 (NH, OH) and 1678 (CO); ^1^H-NMR: 2.03 (s, 3H, CH_3_), 2.35 (s, 3H, CH_3_), 7.20–7.79 (m, 6H, 4 aromatic protons and NH_2_, D_2_O exchangeable), 12.86 (s, 1H, NH, D_2_O exchangeable), 14.53 (s, 1H, hydrogen bonded OH, D_2_O exchangeable); ^13^C-NMR: 13.4 (CH_3_), 72.0 (C-3), 122.8 (C-5), 129.8, 132.2, 134.5, 137.3, 146.0, 160.4 (Ar-H + pyridine C-4, C-6), 172.7 (CO), 185.3 (CS); Mass spectrum *m/z*: 302 (21.6%), 269 (7.5%), 257 (6.5%), 196 (5.8%), 168 (5.8%) and 91 (100.0%); Anal. Calcd for C_14_H_14_N_4_O_2_S: C, 55.61%; H, 4.67%; N, 18.53%; S, 10.61%. Found: C, 55.30%; H, 4.50%; N, 18.40%; S, 10.30%.

*6-Hydroxy-5-(p-methoxyphenylazo)-4-methyl-2-thioxo-1,2-dihydropyridine-3-carbox-amide* (**5c**). Crystallized from dimethylformamide in 91% yield; m.p. 254 °C; IR ν: 3580, 2122 (NH, OH) and 1666 (CO); ^1^H-NMR: 1.98 (s, 3H, CH_3_), 3.85 (s, 3H, OCH_3_), 6.99–7.90 (m, 6H, 4 aromatic protons and NH_2_, D_2_O exchangeable), 12.70 (s, 1H, NH, D_2_O exchangeable), 14.43 (s, 1H, hydrogen bonded OH, D_2_O exchangeable); ^13^C-NMR: 13.6 (CH_3_), 55.8 (OCH_3_), 72.1 (C-3), 120.6 (C-5), 117.4, 132.5, 136.5, 140.3, 156.0, 160.6 (Ar-H + pyridine C-4, C-6), 173.5 (CO), 183.1 (CS); Mass spectrum *m/z*: 318 (16.1%), 274 (22.5%), 242 (9.5%), 183 (5.0%), and 107 (100.0%); Anal. Calcd for C_14_H_14_N_4_O_3_S: C, 52.82%; H, 4.43%; N, 17.60%; S, 10.07%. Found: C, 53.00%; H, 4.70%; N, 17.60%; S, 10.30%.

### 3.4. 3,5-Bis-arylazo-6-hydroxy-4-methyl-2-thioxo-2,3-dihydropyridine-3-carboxamides ***6a–d***

*Route A*: To a cold solution of **5** (0.01 mole) in pyridine (50 mL), containing potassium hydroxide (0.3 g), the arenediazonium chloride (0.01 mole) [prepared by adding concentrated hydrochloric acid (3 mL) to aromatic amine (0.01 mol) at 0 °C and treating the resulting hydrochloride with a cold solution of sodium nitrite (0.69 g, 0.01 mol) in water (5 mL)] was added dropwise with stirring at 0 °C. The coupling mixture was stirred at room temperature for two hours and then diluted with water (30 mL). The resultant crude product thus precipitated was collected by filtration, washed with water, dried and crystallized from the proper solvent.

*3-(p-Chlorophenylazo)-6-hydroxy-4-methyl-2-thioxo-5-(p-tolylazo)-2,3-dihydropyridine-3-carboxamide* (**6a**). According to the general method a cold solution of **5b** (3.02 g, 0.01 mol) in pyridine (50 mL), containing 0.3 g potassium hydroxide, the (*p*-chlorophenyl)diazonium chloride (0.01 mol) [prepared by adding concentrated hydrochloric acid (3 mL) to *p*-chloroaniline (1.27 g, 0.01 mole) at 0 °C and treating the resulting hydrochloride with a cold solution of sodium nitrite (0.69 g, 0.01 mole) in water (5 mL)] was added dropwise with stirring at 0 °C. The coupling mixture was stirred at room temperature for two hours and then diluted with water (30 mL). The resultant crude product thus precipitated was collected by filtration, washed with water, dried and crystallized from ethanol to afford **6a** in 90% yield as a deep reed crystals. m.p. 249 °C; IR ν: 3406–2222 (NH, OH) and 1659 (CO); ^1^H-NMR: 2.09 (s, 3H, CH_3_), 2.63 (s, 3H, CH_3_), 7.26–8.90 (m, 10H, 8 aromatic protons and NH_2_, D_2_O exchangeable), 14.23 (s, 1H, OH, D_2_O exchangeable); ^13^C-NMR: 10.3 (CH_3_), 25.1 (CH_3_), 94.8 (pyridine C-3), 110.6, 122.4, 128.7, 130.0, 131.9, 132.5, 136.5, 138.8, 144.3, 150.0, 160.4 (Ar-H + pyridine C-4, C-5, C-6), 172.3 (CO), 184.1 (CS); Mass spectrum *m/z*: 442 (17.1%), 440 (53.0%), 396 (12.6%), 268 (93.1%), 257 (2.7%), 228 (2.1%) and 91 (100.0%); Anal. Calcd for C_20_H_17_ClN_6_O_2_S: C, 54.48%; H, 3.89; Cl, 8.04; N, 19.06; S, 7.27. Found: C, 54.80%; H, 3.90%; Cl, 7.90%; N, 19.30%; S, 7.30%.

*5-(p-Chlorophenylazo)-6-hydroxy-4-methyl-2-thioxo-3-(p-tolylazo)-2,3-dihydropyridine-3-carboxamide* (**6b**). Crystallized from ethanol in 76% yield; m.p. 259 °C; IR ν: 3375–1909 (NH, OH) and 1659 (CO); ^1^H-NMR: 2.11 (s, 3H, CH_3_), 2.63 (s, 3H, CH_3_), 7.20–8.88 (m, 10H, 8 aromatic protons and NH_2_, D_2_O exchangeable), 14.23 (s, 1H, OH, D2O exchangeable); ^13^C-NMR: 10.5 (CH_3_), 25.1 (CH_3_), 94.2 (pyridine C-3), 110.6, 122.5, 128.6, 130.0, 131.5, 132.3, 136.1, 139.0, 144.3, 150.0, 160.4 (Ar-H + pyridine C-4, C-5, C-6), 172.0 (CO), 184.5 (CS); Mass spectrum *m/z*: 442 (23.9%), 440 (73.1%), 396 (18.7%), 268 (77.1%), 248 (2.1%) and 91 (100.0%); Anal. Calcd for C_20_H_17_ClN_6_O_2_S: C, 54.48; H, 3.89; Cl, 8.04; N, 19.06; S, 7.27. Found: C, 54.60%; H, 3.70%; Cl, 8.20%; N, 19.20%; S, 7.50%. 

*3,5-Bis-(p-tolylazo)-6-hydroxy-4-methyl-2-thioxo-2,3-dihydropyridine-3-carboxamide* (**6c**). Crystallized from ethanol in 96% yield; m.p. 212 °C; IR ν: 3402–2226 (NH, OH) and 1659 (CO); ^1^H-NMR: 1.94 (s, 3H, CH_3_), 2.38 (s, 3H, CH_3_), 2.44 (s, 3H, CH_3_), 7.16–7.80 (m, 10H, 8 aromatic protons and NH_2_, D_2_O exchangeable), 14.30 (s, 1H, OH, D_2_O exchangeable); ^13^C-NMR: 11.1 (CH_3_), 22.1 (CH_3_), 22.8 (CH_3_), 97.2 (pyridine C-3), 110.6, 122.5, 128.6, 129.6, 130.3, 135.3, 136.1, 139.0, 144.3, 148.1, 160.4 (Ar-H + pyridine C-4, C-5, C-6), 171.1 (CO), 184.0 (CS); Mass spectrum *m/z*: 420 (74.4%), 376 (5.4%), 329 (7%), 301 (6.6%), 210 (3.4%) and 91 (100.0%); Anal. Calcd for C_21_H_20_N_6_O_2_S: C, 59.98%; H, 4.79%; N, 19.99%; S, 7.63%. Found: C, 60.10%; H, 4.50%; N, 20.10%; S, 7.80%. 

*3,5-Bis-(p-methoxyphenylazo)-6-hydroxy-4-methyl-2-thioxo-2,3-dihydropyridine-3-carboxamide* (**6d**). Crystallized from ethanol in 87% yield; m.p. 248 °C; IR ν: 3418–2195 (NH, OH) and 1659 (CO); ^1^H-NMR: 2.17 (s, 3H, CH_3_), 3.82 (s, 3H, OCH_3_), 3.88 (s, 3H, OCH_3_), 7.04–8.13 (m, 10H, 8 aromatic protons and 2H, NH_2_, D_2_O exchangeable), 14.17 (s, 1H, OH, D_2_O exchangeable); ^13^C-NMR: 11.5 (CH_3_), 53.1 (OCH_3_), 53.8 (OCH_3_), 92.5 (pyridine C-3), 110.3, 115.3, 123.2, 126.5, 130.1, 133.3, 135.2, 137.0, 155.0, 158.9, 160.2 (Ar-H + pyridine C-4, C-5, C-6), 173.0 (CO), 184.6 (CS); Mass spectrum *m/z*: 452 (3.4%), 424 (66.5%), 408 (10.1%), 379 (13.3%), 273 (2.5%), 229 (22.1%) and 107 (100.0%); Anal. Calcd for C_21_H_20_N_6_O_4_S: C, 55.74%; H, 4.46%; N, 18.57%; S, 7.09%. Found: C, 55.70%; H, 4.30%; N, 18.40%; S, 7.30%. 

*Route B*: To a cold solution of **2** (0.92 g, 0.005 mole) in pyridine (50 mL), containing potassium hydroxide (0.3 g), the arenediazonium chloride (0.01 mole) [prepared by adding concentrated hydrochloric acid (3 mL) to aromatic amine (0.01 mol) at 0 °C and treating the resulting hydrochloride with a cold solution of sodium nitrite (0.69 g, 0.01 mol) in water (5 mL)] was added dropwise with stirring at 0 °C. The coupling mixture was stirred at room temperature for two hours and then diluted with water (30 mL). The resultant crude product thus precipitated was collected by filtration, washed with water, dried and crystallized. Compound **6c** was thus obtained in 93% yield and matched the same compound prepared by Route A in every aspect (m.p., mixed m.p. and IR spectrum). Compound **6d** was thus obtained in 96% yield and matched the same compound prepared by Route A in every aspect (m.p., mixed m.p. and IR spectrum).

### 3.5. 4-Methyl-2,3,6,7-tetrahydroisothiazolo[5,4-b]pyridine-3,6-dione *(**7**)*

To a solution of **2** (1.84 g, 0.01 mole) in ethanol (50 mL), in the presence of potassium hydroxide (0.56 g, 0.01 mole), potassium ferricyanide (2.5 g, 0.01 mol) was added dropwise with stirring at room temperature. The mixture was then stirred at room temperature for two hours and then diluted with cold water (30 mL). The resultant crude product thus precipitated was collected by filtration, washed with water, dried and crystallized from dimethylformamide to afford **7** in 80% yield as a white powder; m.p. 292 °C; IR ν: 3420 (NH) and 1681 and 1636 (2 CO); ^1^H-NMR: 2.42 (s, 3H, CH_3_), 6.11 (s, 1H, pyridine-H5), 11.37 (br.s, 2H, 2 NH, D_2_O exchangeable); ^13^C-NMR: 23.1 (CH_3_), 112.3, 127.1, 131.4, 143.0 (pyridine C atoms), 165.3 (CO), 175.0 (CO); Mass spectrum *m/z*: 182 (27.3%), 154 (100.0%), 107 (14.3%) and 99 (4.8%); Anal. Calcd for C_7_H_6_N_2_O_2_S: C, 46.14%; H, 3.32%; N, 15.38%; S, 17.60%. Found: C, 45.90%; H, 3.50%; N, 15.30%; S, 17.40%. 

### 3.6. 2,2'-(Methylene-bis(sulfanediyl))bis(4-methyl-6-oxo-1,6-dihydropyridine-3-carboxamide) *(**8**)*

*Method A*: To a solution of **2** (1.84 g, 0.01 mol) in ethanol (50 mL) in the presence of potassium hydroxide (0.56 g, 0.01 mol), methylene iodide (0.53 mL, 0.005 mol) was added dropwise with stirring at room temperature. The mixture was then stirred under reflux for two hours and then diluted with cold water (30 mL). The resultant crude product thus precipitated was collected by filtration, washed with water, dried and crystallized from dioxane to afford **8**. *Method B*: The same reactants of method A were heated under microwaves at 500 W and 140 °C for 5 min. The reaction mixture was treated in a similar manner to method A to give compound **8**. Compound **8** was obtained as a white powder in 68% yield (Method A) or 90% yield (Method B); m.p. 304 °C; IR ν: 3358 and 3171 (NH) and 1681, 1635 (2 CO); ^1^H-NMR: 2.22 (s, 6H, 2 CH_3_), 4.90 (s, 2H, CH_2_), 6.27 (s, 2H, pyridine H-5), 7.52–7.77 (br.s, 4H, 2 NH_2_, D_2_O exchangeable), 11.00 (s, 2H, 2 NH, D_2_O exchangeable); ^13^C-NMR: 20.1 (CH_3_), 32.7 (CH_2_), 100.4, 125.1, 143.3, 148.0 (pyridine C atoms), 162.3 (CO), 171.2 (CO); Mass spectrum *m/z*: 380 (1.5%), 336 (2.0%), 321 (1.4%), 197 (29.4%), 183 (4.2) and 53 (100.0%); Anal. Calcd for C_15_H_16_N_4_O_4_S_2_: C, 47.36; H, 4.24%; N, 14.73%; % S, 16.86%. Found: C, 47.20%; H, 4.30%; N, 14.70%; S, 16.80%. 

### 3.7. 2-Hydroxy-5-methyl-4H-pyrido[3,2-e][1,3]thiazine-4,7(8H)-dione *(**10**)*

*Method A*: A mixture of **2** (1.84 g, 0.01 mole) and ethyl chloroformate (0.01 mole) was heated under reflux in absolute ethanol (30 mL) in the presence of sodium ethoxide (0.46 g, 0.02 mole) for 24 h. The reaction mixture was then poured onto water, the deposited precipitate was filtered off, dried and crystallized from ethanol to give **10**. *Method B*: The same reactants of method A were heated under microwaves at 500 W and 140 °C for 30 min. The reaction mixture was treated in a similar manner to method A to give compound **10**. The product was obtained as a white powder in 75% yield (Method A) and 92% yield (Method B); m.p. 205 °C; IR ν: 3410 and 3175 (NH, OH) and 1651 and 1645 (2 CO); ^1^H-NMR: 2.41 (s, 3H, CH_3_), 6.11 (s, 1H, pyridine-H), 8.13 (s, 1H, NH, D_2_O exchangeable), 9.16 (s, 1H, OH, D_2_O exchangeable); ^13^C-NMR: 21.0 (CH_3_), 123.8, 128.0, 134.9, 144.0, 160.1 (thiazine C-2 + pyridine C atoms), 163.0 (CO), 171.6 (CO); Mass spectrum *m/z*: 210 (0.8%), 167 (100.0%), 150 (1.5%), 139 (21.0%) and 107 (21.9); Anal. Calcd for C_8_H_6_N_2_O_3_S: C, 45.71%; H, 2.88%; N, 13.33%; S, 15.25%. Found: C, 45.40%; H, 3.10%; N, 13.10%; S, 15.00%. 

### 3.8. 2-Arylmethylene-8-hydroxy-6-methyl-2,3,4,5-tetrahydropyrido[3,2-f][1,4]thiazepine-3,5-diones ***11a,b***

*Method A*: A mixture of **2** (1.84 g, 0.01 mole), chloroacetic acid (1.2 g, 0.012 mole), the appropriate aromatic aldehyde (0.01 mole) and anhydrous sodium acetate (2 g) was refluxed in glacial acetic acid (30 mL) and acetic anhydride (15 mL) for five hours. The reaction mixture was allowed to cool, poured into water, the deposited precipitated thus formed was filtered off, washed with water, dried and crystallized from the proper solvent. *Method B*: The same reactants of method A were heated under microwaves at 500 w and 140 °C for 10 min. The reaction mixture was treated in a similar manner to method A to give compounds **11a,b**.

*2-(p-Chlorophenylmethylene)-8-hydroxy-6-methyl-2,3,4,5-tetrahydropyrido[3,2-f][1,4]-thiazepine-3,5-dione* (**11a**). Crystallized from dimethylformamide in 73% yield (Method A) and 93% yield (Method B); m.p. 282 °C; IR ν: 3440–2927 (broad, OH and NH), 3024 (CH aromatic), 2927 (CH aliphatic), 1764 and 1670 (2 CO); ^1^H-NMR: 2.35 (s, 3H, CH_3_), 6.25 (s, 1H, pyridine-H), 7.50–7.85 (m, 5H, 4 aromatic protons + methine proton), 7.92 (s, 1H, OH, D_2_O exchangeable), 11.10 (s, 1H, NH, D_2_O exchangeable); ^13^C-NMR: 19.3 (CH_3_), 109.3, 122.0, 124.6, 130.9, 132.0, 134.4, 135.1, 136.9, 146.1, 156.3, 160.1 (pyridine C atoms + thiazepine C-2 + aromatic C atoms + methine C), 167.0 (CO), 171.2 (CO); Mass spectrum *m/z*: 348 (5.3%), 346 (16.7%), 235 (47.1%), 210 (11.5%), 179 (21.0%) and 111 (100.0%); Anal. Calcd for C_16_H_11_ClN_2_O_3_S: C, 55.42%; H, 3.20%; Cl, 10.22%; N, 8.08%; S, 9.25%. Found: C, 55.30%; H, 3.40%; Cl, 10.10%; N, 8.20%; S, 9.40%. 

*8-Hydroxy-2-(p-methoxyphenylmethylene)-6-methyl-2,3,4,5-tetrahydro-pyrido[3,2-f]-[1,4]thiazepine-3,5-dione* (**11b**). Crystallized from dimethylformamide in 71% yield (Method A) and 88% yield (Method B); m.p. 263 °C; IR ν: 3416–2924 (broad, OH and NH), 3068 (CH aromatic), 2924 (CH aliphatic), 1747 and 1664 (2 CO); ^1^H-NMR: 2.40 (s, 3H, CH_3_), 3.90 (s, 3H, OCH_3_), 6.13 (s, 1H, pyridine-H), 6.85–7.75 (m, 5H, 4 aromatic protons + methine proton), 8.11 (s, 1H, OH, D_2_O exchangeable), 11.40 (s, 1H, NH, D_2_O exchangeable); ^13^C-NMR: 18.8 (CH_3_), 108.5, 115.0, 120.1, 130.9, 132.0, 135.4, 136.1, 136.9, 146.1, 156.5, 160.0 (pyridine C atoms + thiazepine C-2 + aromatic C atoms + methine C), 166.8 (CO), 170.4 (CO); Mass spectrum *m/z*: 342 (15.0%), 235 (42.5%), 207 (14.5%), 175 (11.8%) and 107 (100.0%); Anal. Calcd for C_17_H_14_N_2_O_4_S: C, 59.64%; H, 4.12%; N, 8.18%; S, 9.37%. Found: C, 59.80%; H, 4.30%; N, 8.50%; S, 9.40%. 

### 3.9. 2-[(3-Carbamoyl-6-hydroxy-4-methylpyridin-2-yl)thio]acetic Acid *(**12**)*

*Method A*: A mixture of **2** (1.84 g, 0.01 mole), chloroacetic acid (1.2 g, 0.012 mole), and anhydrous sodium acetate (2 g) was refluxed in glacial acetic acid (30 mL) for three hours. The reaction mixture was then allowed to cool, poured into water, the deposited precipitated thus formed was filtered off, washed with water, dried and crystallized from acetic acid to yield **12**. *Method B*: The same reactants of method A were heated under microwaves at 500 W and 140 °C for 5 min. The reaction mixture was treated in a similar manner to method A to give compound **12**. The product was obtained as purple crystals in 74% yield (Method A) and 92% yield (Method B); m.p. 224 °C; IR ν: 3375–2473 (NH, OH) and 1724 & 1651 (2 CO); ^1^H-NMR: 2.48 (s, 3H, CH_3_), 4.10 (s, 2H, CH_2_), 5.90 (s, 1H, pyridine-H), 10.23 (s, 1H, OH, D_2_O exchangeable), 11.10 (s, 2H, 2 NH, D_2_O exchangeable), 11.60 (s, 1H, COOH, D_2_O exchangeable); ^13^C-NMR: 20.1 (CH_3_), 32.5 (CH_2_), 111.2, 115.4, 142.0, 153.6, 154.0 (pyridine C), 168.5 (CO), 173.9 (CO); Mass spectrum *m/z*: 242 (8.5%), 197 (51.5%), 151 (78.5%), 107 (11.0%) and 45 (100.0%); Anal. Calcd for C_9_H_10_N_2_O_4_S: C, 44.62%; H, 4.16%; N, 11.65%; S, 13.24%. Found: C, 44.70%; H, 4.40%; N, 11.80%; S, 13.10%.

### 3.10. 8-Hydroxy-6-methyl-2,3,4,5-tetrahydropyrido[3,2-f][1,4]thiazepine-3,5-dione *(**13**)*

*Method A*: A mixture of **2** (1.84 g, 0.01 mole), chloroacetic acid (1.2 g, 0.012 mole), and anhydrous sodium acetate (2 g) in glacial acetic acid/acetic anhydride (30 mL/15 mL) was heated in a boiling water bath for three hours. The reaction mixture was then allowed to cool, poured into water, the deposited precipitate thus formed was filtered off, washed with water, dried and crystallized from acetic acid to afford **13**. *Method B*: The same reactants of method A were heated under microwaves at 500 W and 140 °C for 5 min. The reaction mixture was treated in a similar manner to method A to give compound **13**. Product **13** was obtained as a brown powder in 43% yield (Method A) and 66 % yield (Method B); m.p. 258 °C; IR ν: 3400 and 3314 (NH, OH) and 1774 and 1678 (2 CO); ^1^H-NMR: 2.50 (s, 3H, CH_3_), 3.95 (s, 2H, CH_2_), 6.04 (s, 1H, pyridine-H), 9.31 (s, 1H, NH, D_2_O exchangeable), 11.55 (s, 1H, OH, D_2_O exchangeable); ^13^C-NMR: 19.4 (CH_3_), 38.9 (CH_2_), 110.0, 114.3, 140.1, 154.3, 155.0 (pyridine C), 167.4 (CO), 170.0 (CO); Mass spectrum *m/z*: 224 (17.0%), 196 (34.7%), 181 (10.3%), 153 (3.6%) and 107 (100.0%); Anal. Calcd for C_9_H_8_N_2_O_3_S: C, 48.21%; H, 3.60%; N, 12.49%; S, 14.30%. Found: C, 48.40%; H, 3.50%; N, 12.30%; S, 14.10%.

### 3.11. 7-Arylazo-2-(p-methoxyphenylmethylene)-8-hydroxy-6-methyl-2,3,4,5-tetrahydropyrido[3,2-f]-[1,4]thiazepine-3,5-diones ***14a–c***

To a cold solution of **11b** (3.42 g, 0.01 mole) in pyridine (50 mL), containing potassium hydroxide (0.3 g), the arenediazonium chloride (0.01 mole) [prepared by adding concentrated hydrochloric acid (3 mL) to aromatic amine (0.01 mole) at 0 °C and treating the resulting hydrochloride with a cold solution of sodium nitrite (0.69 g, 0.01 mole) in water (5 mL)] was added dropwise with stirring at 0 °C. The coupling mixture was then stirred at room temperature for two hours and then diluted with water (30 mL). The resultant crude product thus precipitated was collected by filtration, washed with water, dried and crystallized from the proper solvent.

*7-(p-Chlorophenylazo)-2-(p-methoxyphenylmethylene)-8-hydroxy-6-methyl-2,3,4,5-tetrahydropyrido-[3,2-f][1,4]thiazepine-3,5-dione* (**14a**). Crystallized from dilute dimethyl-formamide in 63% yield; m.p. 310 °C; IR ν: 3480–2100 (NH, OH) and 1740 and 1670 (2 CO); ^1^H-NMR: 2.45 (s, 3H, CH_3_), 3.88 (s, 3H, OCH_3_), 6.90–7.81 (m, 9H, 8 aromatic protons + methine proton), 9.23 (s, 1H, NH, D_2_O exchangeable), 11.00 (s, 1H, OH, D_2_O exchangeable); ^13^C-NMR: 13.4 (CH_3_), 57.0 (OCH_3_), 109.0, 115.3, 124.3, 127.1, 127.9, 128.8, 129.7, 131.2, 135.1, 136.0, 142.9, 145.0, 156.4, 160.2, 161.3 (pyridine C atoms + thiazepine C-2 + aromatic C atoms + methine C), 169.0 (CO), 173.7 (CO); Mass spectrum *m/z*: 482 (3.5%), 480 (11.3%), 373 (28.8%), 341 (5.7%) and 107 (100.0%); Anal. Calcd for C_23_H_17_ClN_4_O_4_S: C, 57.44%; H, 3.56%; Cl, 7.37%; N, 11.65%; S, 6.67%. Found: C, 57.60%; H, 3.30%; Cl, 7.50%; N, 11.70%; S, 6.90%.

*7-(p-Methoxyphenylazo)-2-(p-methoxyphenylmethylene)-8-hydroxy-6-methyl-2,3,4,5-tetrahydropyrido-[3,2-f][1,4]thiazepine-3,5-dione* (**14b**). Crystallized from ethanol in 72% yield; m.p. 332 °C; IR ν: 3454–2218 (NH, OH) and 1751 and 1666 (2 CO); ^1^H-NMR: 2.52 (s, 3H, CH_3_), 3.85 (s, 3H, OCH_3_), 3.95 (s, 3H, OCH_3_), 6.94–7.74 (m, 9H, 8 aromatic protons + methine proton), 9.15 (s, 1H, NH, D_2_O exchangeable), 11.10 (s, 1H, OH, D_2_O exchangeable); ^13^C-NMR: 12.8 (CH_3_), 56.9 (OCH_3_), 57.9 (OCH_3_), 108.0, 114.3, 115.2 122.1, 125.6, 127.1, 129.8, 130.7, 135.1, 142.9, 145.0, 156.4, 159.1, 160.3, 163.8 (pyridine C atoms + thiazepine C-2 + aromatic C atoms + methine C), 168.3 (CO), 170.3 (CO); Mass spectrum m/z: 476 (15.1%), 369 (31.1%), 341 (5.2%), 273 (10.4%) and 107 (100.0%); Anal. Calcd for C_24_H_20_N_4_O_5_S: C, 60.49%; H, 4.23%; N, 11.76%; S, 6.73%. Found: C, 60.60%; H, 4.10%; N, 11.70%; S, 6.90%.

*7-(p-Tolylazo)-2-(p-methoxyphenylmethylene)-8-hydroxy-6-methyl-2,3,4,5-tetrahydro-pyrido[3,2-f]-[1,4]thiazepine-3,5-dione* (**14c**). Crystallized from ethanol in 66% yield; m.p. 325 °C. IR ν: 3414–2213 (NH, OH) and 1753 and 1661 (2 CO). ^1^H-NMR: 2.30 (s, 3H, CH_3_), 2.43 (s, 3H, CH_3_), 3.85 (s, 3H, OCH_3_), 6.90–7.80 (m, 9H, 8 aromatic protons + methine proton), 9.41 (s, 1H, NH, D_2_O exchangeable), 11.22 (s, 1H, OH, D_2_O exchangeable); ^13^C-NMR: 11.0 (CH_3_), 21.9 (CH_3_), 55.9 (OCH_3_), 108.8, 113.7, 115.1 124.1, 125.3, 126.3, 128.8, 130.0, 135.1, 142.6, 144.1, 155.2, 157.0, 160.3, 162.9 (pyridine C atoms + thiazepine C-2 + aromatic C atoms + methine C), 168.0 (CO), 171.7 (CO); Mass spectrum *m/z*: 460 (17.5%), 353 (25.5%), 341 (6.6%), 253 (5.4%) and 107 (100.0%). Anal. Calcd for C_24_H_20_N_4_O_4_S: C, 62.60%; H, 4.38%; N, 12.17%; S, 6.96%. Found: C, 62.40%; H, 4.50%; N, 12.10%; S, 7.10%.

## 4. Conclusions

Several new isothiazolopyridines, pyridothiazines and pyridothiazepines have been synthesized using both traditional methods and microwave assisted conditions. The latter methods proved much more efficient in reducing reaction times as well as increasing the overall yield of the reactions. Structures of the newly synthesized compounds were proven by both spectral and chemical methods.
